# Caspase-1 genetic variation is not associated with Alzheimer's disease risk

**DOI:** 10.1186/1471-2350-11-32

**Published:** 2010-02-25

**Authors:** José Luis Vázquez-Higuera, Eloy Rodríguez-Rodríguez, Pascual Sánchez-Juan, Ignacio Mateo, Ana Pozueta, Ana Martínez-García, Ana Frank, Fernando Valdivieso, José Berciano, María J Bullido, Onofre Combarros

**Affiliations:** 1 Neurology Service and CIBERNED, "Marqués de Valdecilla" University Hospital (University of Cantabria), Santander, Spain; 2 Molecular Biology Department and CIBERNED, Centro de Biología Molecular Severo Ochoa (CSIC-UAM), Madrid, Spain; 3 Neurology Service and CIBERNED, Hospital Universitario La Paz (UAM), Madrid, Spain

## Abstract

**Background:**

Interleukin (IL)-1β is a potent proinflammatory cytokine markedly overexpressed in the brains of patients with Alzheimer's disease (AD), and also involved in development of atherosclerosis and coronary artery disease. Caspase-1 (CASP1), formerly called IL-1β converting enzyme (ICE), mediates the cleavage of the inactive precursor of IL-1β into the biologically active form. CASP1 genetic variation (G+7/in6A, rs501192) has been associated with susceptibility to myocardial infarction and cardiovascular death risk. We examined the contribution of this gene to the susceptibility for AD.

**Methods:**

We examined genetic variations of CASP1 by genotyping haplotype tagging SNPs (htSNPs) (rs501192, rs556205 and rs530537) in a group of 628 Spanish AD cases and 722 controls.

**Results:**

There were no differences in the genotypic, allelic or haplotypic distributions between cases and controls in the overall analysis or after stratification by age, gender or APOE ε4 allele.

**Conclusion:**

Our negative findings in the Spanish population argue against the hypothesis that CASP1 genetic variations are causally related to AD risk.

## Background

A chronic inflammatory process might contribute to the neurodegeneration associated with Alzheimer's disease (AD), by overexpression of cytokines, such as interleukin-1 (IL-1), and other inflammatory molecules in activated microglia surrounding amyloid plaques [1]. Increased expression of IL-1 in AD has been implicated in the formation of amyloid plaques and neurofibrillary tangles, the spread of these neuropathological lesions across cerebral cortical regions, and the accompanying neuronal cell injury and loss [2]. The predominant β isoform of IL-1 is generated from an inactive precursor through the action of caspase-1 (CASP1), a cystein protease formerly called IL-1β converting enzyme (ICE), and CASP1 expression appears to be significantly increased in post-mortem brain tissue from patients with AD [3-5]. In addition, CASP1 messenger RNA expression has been closely associated with neurofibrillary tangle and, to a lesser extent, amyloid plaque density [4]. Several studies have reported associations between IL-1β genetic polymorphisms and AD, but findings from different studies have been controversial [6]. Although genetic markers of the CASP1 region were not found associated to AD in recent genome-wide association studies [7-10], Blankenberg et al. [11] sequenced the CASP1 gene in a case-control study of myocardial infarction (MI), and found that CASP1 genetic variation is associated with cardiovascular risk. The present study investigated the genetic variability of the CASP1 in relation to AD risk.

## Methods

The study included 628 AD patients (65% women; mean age at study 75.8 years; SD 8.1; range 61-109 years; mean age at onset 72.7 years; SD 7.9; range 60-108 years) who met NINCDS/ADRDA criteria for probable AD [12]. All AD cases were defined as sporadic because their family history did not mention any first-degree relative with dementia. AD patients were recruited from the Departments of Neurology of University Hospital "Marqués de Valdecilla" (Santander, Spain), and Hospital "La Paz" (Madrid, Spain). The large majority of patients were living in the community and had been referred by their general practitioner; few had been admitted from hospital wards or nursing home facilities. Control subjects were 722 unrelated individuals (65% women; mean age 78.7 years; SD 9.3; range 60-104 years) randomly selected from nursing homes. These subjects had complete neurologic and medical examinations that showed that they were free of significant illness and had Mini Mental State Examination scores of 28 or more, which were verified by at least one subsequent annual following-up assessment. The controls arose from the same base population as the cases. The AD and control samples were Caucasians originating from a limited geographical area in northern Spain (Santander) and from the central area of Spain (Madrid).

Blood samples were taken after written informed consent had been obtained from the subjects or their representatives. The study was approved by the ethical committees of the University Hospital "Marqués de Valdecilla" and the Hospital "La Paz". Genotyping of CASP1 (rs501192 in intron 6, rs556205 in intron 7, and rs530537 in intron 7) polymorphisms was performed by a Taq-Man single-nucleotide-polymorphism assay (Applied Biosystems, Warrington, Cheshire, UK) and an ABI PRISM 7000 or 7900 HT sequence detection systems (Applied Biosystems). We used data from the HapMap project http://www.hapmap.org to select the 3 htSNPs capturing 85% of CASP1 genetic variability in Caucasians. SNPs were chosen among those with minor allele frequencies ≥ 5% using Haploview v3.2 software http://www.broad.mit.edu/mpg/haploview with an r^2 ^threshold of 0.8. APOE genotyping was performed by amplification of the 4th exon of the APOE gene by PCR with biotinylated primers, followed by reverse hybridization on nitrocellulose strips, using the INNO-LIPA ApoE assay (Innogenetics NV, Ghent, Belgium), or by HhaI restriction analysis.

Hardy-Weinberg equilibrium (HWE) was calculated for the 3 htSNPs genotypes in the control population using Pearson's χ^2 ^statistics. We assessed pairwise linkage disequilibrium (LD) between the 3 htSNPs by D' and r^2 ^statistics. Haplotype reconstruction and their frequencies in cases and controls were estimated by an expectation-maximization algorithm. Pearson's χ^2 ^statistics were performed to compare allele distribution of the patients and control for each htSNP. Haplotype frequencies were also assessed using Pearson's χ^2 ^using Haploview 3.32 software http://www.broad.mit.edu/mpg/haploview. Rare haplotypes (total frequency < 0.05) were excluded from the analysis.

## Results

In control groups, no significant deviations from Hardy-Weinberg equilibrium were found for any of the 3 SNPs (p-values ranging from 0.20 to 0.96). As shown in Table 1, the distribution of the allele and genotype frequencies of the CASP1 htSNPs did not differ significantly between AD and control groups. The three CASP1 htSNPs were in complete LD with each other (with r^2^values close to 1.0) forming one block, and therefore, they exhibited a pattern of reduced haplotype diversity, with only four haplotypes captured that did not differ between AD and control groups (Table 2). Our analysis showed the expected association between the APOE ε4 allele and AD with an OR = 5.92 (95% CI = 4.60-7.62, p < 0.001) for carrying 1 or 2 copies of ε4 allele. There were no major differences in allele, genotype or haplotype frequencies of CASP1 polymorphisms in our total sample stratified for age, gender or APOE ε4 allele.

**Table 1:** Distribution of caspase-1 polymorphisms in patients and controls

Caspase-1 polymorphisms	Patients	Controls
rs501192 (intron 6)		
GG	436 (0.72)	519 (0.73)
GA	151 (0.25)	175 (0.25)
AA	18 (0.03)	15 (0.02)
Total	605	709
Allele frequency G/A	0.84/0.16	0.85/0.15
		
rs556205 (intron 7)		
AA	514 (0.82)	606 (0.84)
AC	109 (0.17)	113 (0.16)
CC	5 (0.01)	3 (0.00)
Total	628	722
Allele frequency A/C	0.90/0.10	0.92/0.08
		
rs530537 (intron 7)		
AA	159 (0.26)	200 (0.28)
AG	313 (0.52)	374 (0.52)
GG	134 (0.22)	144 (0.20)
Total	606	718
Allele frequency A/G	0.52/0.48	0.54/0.46

Figures in parentheses indicate frequencies; p-values > 0.05 for all allelic and genotypic comparisons

**Table 2:** Haplotype association analysis between caspase-1 gene and AD

Haplotype block	Haplotype frequency	AD, control frequency	p-value
GAA	0.531	0.521, 0.540	0.32
GAG	0.319	0.324, 0.315	0.60
ACG	0.088	0.094, 0.082	0.29
AAG	0.062	0.061, 0.063	0.83

Haplotype block consists of SNPs: rs501192, rs556205 and rs530537; rare haplotypes (total frequency < 0.05) were excluded from the analysis; p-values were not corrected for multiple comparisons.

## Discussion

Increasing evidence indicates that IL-1 is involved in both development of atherosclerosis and cardiovascular risk, and CASP1 might play a key role in the proatherogenic effects mediated by IL-1β [11]. Blankenberg et al. [11] investigated whether polymorphisms of the CASP1 gene might influence cardiovascular risk by analyzing cases with myocardial infarction (MI) and controls in British, French and German populations (n = 1774). They sequenced all exons, up to 100 bp of exon/intron junctions and up to 1 kb of 3'- and 5'-flanking sequences in CASP1, and found a block of 14 SNPs in complete LD covering the whole gene sequence: the CASP1 (G+7/in6A, rs501192) A allele was associated with a lower risk of MI in the British and German populations, and the same allele exhibited a borderline association with prospective cardiovascular death in the German population. Moreover, the CASP1 haplotype carrying the (G+7/in6A, rs501192) A allele was associated with a lower gene expression, supporting the existence of a functional polymorphism within the CASP1 gene. All the 3 tagging SNPs analyzed in our study are located in a single block that is the same haplotype block as described by Blankenberg et al. [11] in their European cohorts. Our main objective was to study the CASP1 rs501192 in intron 6 (G+7/in6A) suggesting CASP1 as a putative gene causing cardiovascular risk, and in addition, we genotyped other SNPs in intron 7 (rs556205 and rs530537). We failed to observe any allele, genotype or haplotype association with AD either in the whole study or in APOE ε4-stratified subgroups in the Spanish population.

Variation in the CASP1 gene has been associated with cognitive function in elderly individuals with normal cognition [13]: subjects carrying the CASP1 rs580253 (exon 6) A allele and rs554344 (3'-UTR) C allele had significantly lower IL-1β production levels and performed better on all executive function tests at baseline and during follow-up compared to homozygous of the wild-type allele. One might postulate that carriers of these CASP1 genetic polymorphisms associated with better memory have a higher "cognitive reserve", and consequently, would have a lower predisposition to AD. Although both of these polymorphisms are in complete linkage disequilibrium with our studied CASP1 genetic variants, we did not find any genetic association with AD risk. Contradictory association of a memory-related genetic polymorphism with either healthy volunteers or AD patients is already well known; for example, the APOE ε4 allele is the only firmly established genetic susceptibility factor for sporadic AD, but it has been related to better memory in young, healthy volunteers [14]. Moreover, healthy young, middle aged and elderly carriers of the KIBRA rs17070145 T-allele exhibited a clear advantage in delayed episodic recall compared to individuals lacking the T-allele [15,16], but a further study in a community AD population showed an association of the KIBRA rs17070145 T-allele with an increase AD risk [17], thus resulting inconsistent with previous findings in healthy subjects. Our negative results are probably not due to insufficient statistical power, because our sample size had enough power (86%) to detect an odds ratio of 1.5 at disease allele frequencies of 0.08. Because we only studied three htSNPs capturing 85% of CASP1 genetic variability in Caucasians, it might be argued that we have missed a hypothetical disease locus, which would have been detected by analysis of extended haplotypes. However, the complete LD across the CASP1 region in our study argues against this possibility; of note, two recent series in other Caucasian populations [11,13] have also detected a complete LD covering the whole gene sequence. Despite supporting evidence for the biological role of CASP1 in AD exists, according to our data CASP1 genetic variation does not seem to be a risk factor for AD.

## Conclusion

Our negative findings in the Spanish population argue against the hypothesis that CASP1 genetic variations are causally related to AD risk.

## Competing interests

The authors declare that they have no competing interests.

## Authors' contributions

JLVH and ERR performed the genetic studies and reviewed critically the manuscript. PSJ performed the statistical analyses and reviewed critically the manuscript. IM, AP, AMG, AF, FV, JB and MJB reviewed critically the manuscript. OC drafted the manuscript and contributed to its final version. All authors read and approved the final manuscript.

## Pre-publication history

The pre-publication history for this paper can be accessed here:

http://www.biomedcentral.com/1471-2350/11/32/prepub
